# Paraneoplastic Manifestation of Primary Squamous Cell Cancer of the Thyroid Gland

**DOI:** 10.7759/cureus.39415

**Published:** 2023-05-23

**Authors:** Anusha Sharma.P.S, Ranjith J, Murali Mohan B.V, Subramanian Kannan

**Affiliations:** 1 Internal Medicine, Narayana Hrudayalaya, Bengaluru, IND; 2 Endocrinology Diabetes and Metabolism, Narayana Hrudayalaya, Bengaluru, IND

**Keywords:** thyroid malignancy, triple endoscopy, anaplastic thyroid cancer, pnm, hypercalcemia, paraneoplastic leukemoid reaction, primary squamous cell carcinoma of thyroid, pscct, squamous cell cancer of the thyroid gland, paraneoplastic manifestation

## Abstract

Paraneoplastic manifestation (PNM) of cancers is a non-metastatic, non-invasive systemic effect of malignancies due to chemokines and hormones produced by the primary neoplasm. Squamous cell cancers (SCCs) are known to present with PNM. Primary SCC of thyroid accounts for <1% of all thyroid malignancies and carries a very poor prognosis. We present a rare case of SCC arising from the thyroid gland who presented with fever, leukemoid reaction and hypercalcemia as part of PNM. A 67-year-old male patient presented with two months history of intermittent high-grade fever, weakness, loss of weight and appetite. Examination revealed a large (~10 cm) hard swelling over the right side of the neck. Investigations revealed neutrophilic leukocytosis, elevated C-reactive protein (CRP) and procalcitonin and hypercalcemia with a normal thyroid-stimulating hormone (TSH). The fever workup was negative for infection. Fine-needle aspiration cytology (FNAC) and core biopsy of the thyroid mass revealed malignant cells with squamous differentiation. An extensive search for possible other primary was ruled out by triple endoscopy. The combination of fever, neutrophilic leukocytosis, hypercalcemia and squamous malignancy was consistent with a diagnosis of PNM of SCC. A fluorodeoxyglucose-positron emission tomography (FDG-PET) CT scan showed a heterogeneously enhancing mass lesion in the right lobe of the thyroid with some retrosternal extension. He underwent total thyroidectomy with bilateral central compartment neck dissection. Final histopathology revealed moderately differentiated SCC of the thyroid. Concurrent chemoradiation was given. Despite continued chemotherapy, he succumbed to illness within six months of diagnosis. Primary SCC of thyroid (PSCCT) is a rare malignancy. It is a highly aggressive tumor having a poor prognosis with a median survival time of about 9-12 months and less clearly defined therapy due to its rarity. Paraneoplastic manifestation of PSCCT is known. As fever, leukemoid reaction and hypercalcemia can be a paraneoplastic manifestation, one should think of PSCCT.

## Introduction

Primary squamous cell carcinoma of thyroid (PSCCT) is a rare and aggressive malignancy. It accounts for <1% of all thyroid malignancies and carries a very poor prognosis [1]. It usually presents as enlarging mass in the neck region with compression symptoms. Squamous cell cancers (SCCs) are known to present with paraneoplastic manifestation (PNM) [2]. PNM of cancers is a non-metastatic, non-invasive systemic effect of malignancies due to chemokines and hormones produced by the primary neoplasm. We present a rare case of SCC arising from the thyroid gland, where the patient presented with fever, leukemoid reaction and hypercalcemia as part of PNM.

## Case presentation

A 67-year-old male patient, with a known case of type 2 diabetes mellitus and systemic hypertension for 11 years and on regular treatment, presented with complaints of intermittent high-grade fever (102°F) for two months, generalized weakness, loss of appetite and weight of about 5 kg in one month. He gave a history of travel (from Bangalore to Delhi for an event, during the COVID-19 outbreak) and developed symptoms of fever. He was treated with multiple courses of oral antibiotics (Tab. amoxicillin clavulanate, Tab. azithromycin). There was no improvement in symptoms. He was newly diagnosed with benign prostatic hyperplasia for a month, and he was started on treatment for the same. He had persistently increased total counts (from 17,000/µL to 25,000/µL to 35,000/µL). After two days of admission, he also gave complaints of a change in his voice.

Physical examination

His heart rate was 84 beats/minute, and BP was 120/80 mmHg. He had pallor. Examination revealed a large (~10 cm) hard swelling over the right side of the neck extending from the angle of the mandible to the sternal notch and no clear inferior border. There were no palpable lymph nodes. Another systemic examination was unremarkable.

Investigations

Investigations revealed anemia (8.3 g/dL), leukocytosis (51,000/µL with 87.8% neutrophils, progressed till 56,600/µL ), thrombocytosis (835 thous/µL), elevated C-reactive protein (CRP) (145.626 mg/l) and procalcitonin (6.31 ng/mL), deranged liver function and hypercalcemia (corrected calcium 11 mg/dL) with a normal thyroid-stimulating hormone (TSH) (2.67 µIU/mL). Parathormone levels were 5.30 pg/mL (13.6-85.8 pg/mL). As a part of pyrexia of unknown origin, he was worked up for malarial, typhoid, Rickettsial and Leptospira infections, which turned negative. Blood and urine cultures were sterile. Peripheral smear was suggestive of normocytic normochromic anemia with neutrophilic leukocytosis and thrombocytosis. Anemia workup showed mean corpuscular volume (MCV) 80.3 fl, mean corpuscular hemoglobin (MCH) 25.90 pg, mean corpuscular hemoglobin concentration (MCHC) 32.3%, serum ferritin of 1220 ng/mL, total iron 22 µg/dl, serum total iron-binding capacity (TIBC) 160 µg/dl, serum transferrin 104 mg/dl and transferrin saturation 13.75%. Ultrasound of the abdomen and pelvis showed hepatomegaly with prostatomegaly. Bone marrow examination showed hypercellular aspirate with a mild predominance of mature plasma cells and no other significant abnormality. Serum protein electrophoresis showed hypoalbuminemia with no paraproteinemia. Serial monitoring of white blood cell counts showed an increasing trend. Ultrasound of the neck was suggestive of a heterogenous mass occupying the whole of the right side of the neck with retrosternal extension.

**Figure 1:**
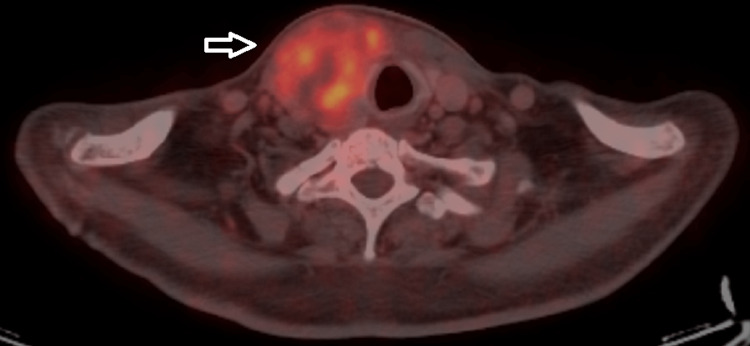
FDG-PET CT showing hypermetabolic heterogeneously enhancing mass lesion in the right lobe of the thyroid (arrow) FDG: fluorodeoxyglucose, PET: positron emission tomography.

A fluorodeoxyglucose (FDG)-positron emission tomography (PET) computed tomography (CT) scan (Figure 1) showed a hypermetabolic heterogeneously enhancing mass lesion in the right lobe of thyroid (size ~5.8 x 5.4 x 6.8 cm SUV max 10.4), which is medially abutting the right lamina of thyroid cartilage extending to sternal notch. Heterogeneously enhancing non-FDG avid retrosternal extension of the above mass lesion in the anterior mediastinum (size 4.8 x 6.7 cm), abutting the right subclavian artery and arch of the aorta. Hepatomegaly (16.4 cm) was noted; there was no FDG uptake in the liver.

**Figure 2:**
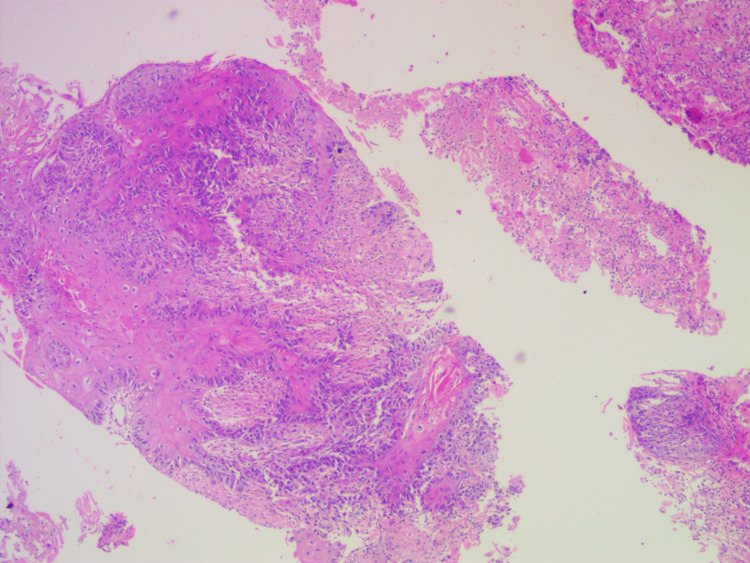
Histopathological examination: cores are infiltrated with squamous cell carcinoma with areas of necrosis

Fine-needle aspiration cytology (FNAC) (Figure 2) smears were highly cellular and showed sheets of atypical squamoid-appearing cells with background necrosis and cystic changes. It was suggestive of carcinoma with squamous differentiation (Bethesda VI). A core biopsy of the thyroid mass revealed malignant cells with squamous differentiation. Possibilities of metastasis of unknown primary to cervical lymph nodes, infiltrating thyroid vs primary squamous cell carcinoma of thyroid (PSCCT), were considered.

The combination of fever, neutrophilic leukocytosis, hypercalcemia and squamous malignancy was consistent with a diagnosis of PNM of SCC. Thrombocytosis was considered multifactorial: elevated serum ferritin levels were suggestive of inflammation, which could be possibly due to malignancy, and also reactive thrombocytosis could be secondary to iron deficiency anemia. A detailed workup was done with triple endoscopy to rule out the other causes of possible primary neoplasm. Bronchoscopy showed a mild bulge in the right side of the trachea, and the rest was unremarkable. Video laryngeal stroboscopy was suggestive of bilaterally normal vocal cords and was mobile, and the pyriform sinus was not visualized; the right aryepiglottic fold was edematous. Upper GI endoscopy showed normal mucosal study and a lax lower esophageal sphincter. However, it did not reveal another primary lesion, and hence, a final diagnosis of SCC of the thyroid was considered.

After a multidisciplinary meeting, it was decided to go ahead with surgery even with borderline resection followed by chemotherapy. Fever and leukocytosis responded to naproxen, and calcium improved with zoledronic acid. He underwent total thyroidectomy with bilateral central compartment neck dissection with right level III and IV lymph node dissection with resection of an internal jugular vein with mediastinal lymph node dissection. The tumor was extremely aggressive and adherent to common carotid, so R2 resection was done. (R0 resection corresponds to resection for the cure or complete remission, and R1 resection amounts to the microscopic residual tumor, whereas R2 resection amounts to the macroscopic residual tumor.) After surgery, he was fever free without antipyretics; leukocytosis and thrombocytosis were resolved.

**Figure 3:**
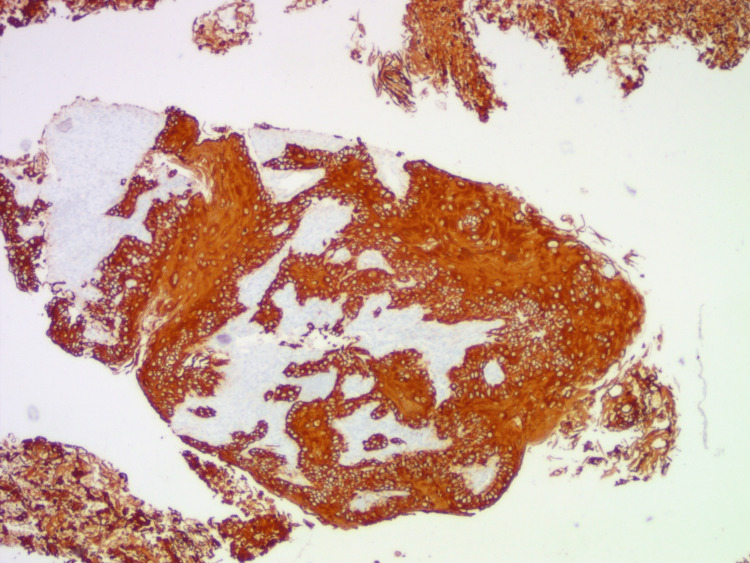
IHC: tumor cells express pan cytokeratin IHC: immunohistochemistry.

**Figure 4:**
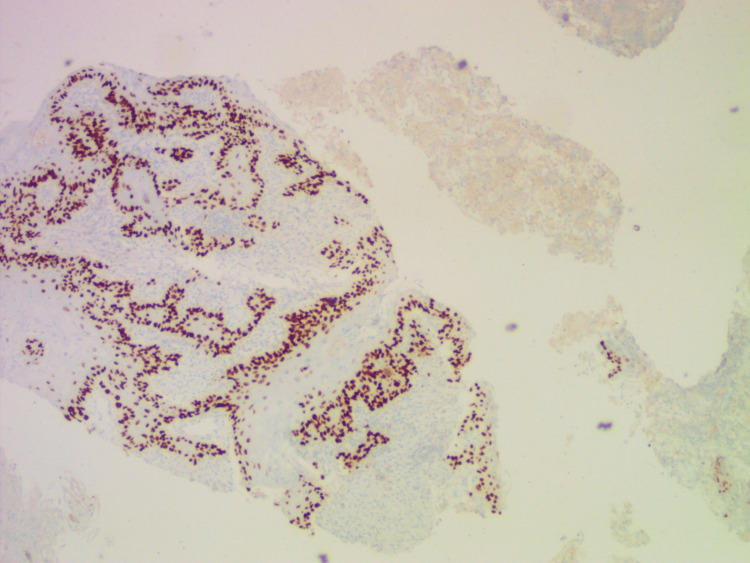
IHC: tumor cells express p63 IHC: immunohistochemistry.

Final histopathology revealed moderately differentiated SCC of the thyroid. Immunohistochemistry (IHC) expressed pan cytokeratin (CK) and p63 and lacked leukocyte common antigen (LCA) and paired-box gene 8 (PAX8) (Figures 3, 4). It was a stage IV disease and had R2 resection. Hence after a multidisciplinary meeting, it was decided to start palliative chemotherapy. Initially, he was started on weekly cisplatin (60 mg for six weeks) regimen with concurrent chemoradiation (70Gy).

Follow-up

He was discharged after 15 days post-surgery and continued his chemotherapy for three months. He had progressive disease despite treatment and was started on palliative chemotherapy (carboplatin and 5-fluorouracil). He was readmitted after three months with complaints of fever and breathing difficulty. Pus culture from the tracheostomy site showed *Enterobacter cloacae* and was treated with antibiotics, but he had a refractory septic shock and cardiac arrest. He succumbed to his illness within six months of diagnosis of PSCCT.

## Discussion

Primary squamous cell carcinoma of thyroid (PSCCT) is a highly aggressive rare tumor (<1%), having a poor prognosis with a median survival time of about 9-12 months and less clearly defined therapy due to its rarity [1]. Fewer cases have been reported in the literature. It represents <1% of all primary carcinomas of the thyroid gland [2].

The development of squamous cells within the thyroid gland is explained with three possible theories: an embryonic origin, metaplastic transformations and dedifferentiation of preexisting thyroid malignancy. It can also be a denovo origin from the follicular epithelium [3]. The observation shows that up to 40% of papillary and many anaplastic thyroid cancers contain patchy regions of the squamous cell population [4]. The mean age at the diagnosis of PSCCT is 63 years, and the women:men ratio is 2:1 [3,4]. PSCCT is predominant in female patients in the fifth or sixth decade of life [5-8].

Patients with PSCCT most commonly present with an enlarging anterior neck mass (70% with extrathyroidal extension, 48.3% with regional lymph node involvement) [3], followed by dyspnea or dysphagia (20%) and change of voice (15%) [5]. Paraneoplastic manifestation in the form of hypercalcemia and leukocytosis in squamous cell carcinoma is rare and has been reported in anaplastic thyroid carcinoma as well as squamous cell carcinomas of the lung and aerodigestive tract. The features were due to the parathyroid hormone-related protein and granulocyte colony-stimulating factor [2].

FNAC has a very limited role in diagnosing squamous cell carcinoma (SCC) of the thyroid [5]. It was also noted that high-grade FNAC findings in thyroid malignancies may raise suspicion for primary SCC of the thyroid [5]. In our case, smears were highly cellular and showed sheets of atypical squamoid-appearing cells with background necrosis and cystic changes. Differential diagnosis includes anaplastic thyroid carcinoma, metastasis from adjacent organs and carcinoma showing thymus-like elements (CASTLE) disease of the thyroid gland [7]. Squamous cell carcinoma of the thyroid is mostly due to secondary metastasis; hence, an extensive search for the possible primary was done in our case with triple endoscopy.

Immunohistochemistry (IHC) remains the key to diagnosis. Expression of CK7, CK19 and p63 is a feature of primary squamous cell carcinoma of the thyroid. Initially, it was proposed that positive PAX8 immunostaining may differentiate primary SCC from secondary SCC where it was usually negative [3]. As most of the thyroid carcinomas express PAX8, details of expression in PSCCT were not clear and it was reported that pure SCC of the thyroid would lack PAX8 expression [4,9]. However, in our patient, PAX8 was negative. As per the latest WHO classification, primary squamous cell carcinoma of the thyroid has been classified as a morphologic pattern of anaplastic thyroid carcinoma due to the presence of human gene encoding protein called B-raf (BRAF) V600E mutations in 60% of cases and has the same prognosis as that of anaplastic carcinoma [10].

Because of the rarity of the disease, a defined treatment with chemoradiation is lacking [6]. PSCCT is poorly responsive to radiotherapy and relatively resistant to chemotherapy. The carcinoma does not uptake iodine. Hence, the role of radioactive iodine ablation or thyroid suppression is not beneficial. The patients being treated with surgery with complete resection alone appear to have the best survival rate [2]. Lenvatinib, a multi-receptor tyrosine kinase inhibitor, has also been evaluated and proposed for the treatment of anaplastic thyroid cancer (ATC), recurrent and metastatic disease. Immunotherapy alone or in combination with other therapy has also been tried in metastatic disease. Treatment response depends on the age at presentation, tumor burden and metastasis [2,4].

## Conclusions

Primary SCC of thyroid (PSCCT) is an extremely rare and aggressive malignancy. The origin of squamous cells in the thyroid has been proposed by various theories. Paraneoplastic manifestation of PSCCT is known. Paraneoplastic manifestation in the form of fever, leukemoid reaction and hypercalcemia can be due to the parathyroid hormone-related protein and granulocyte colony-stimulating factor. Immunohistochemistry (IHC) plays an important role in the diagnosis. Latest WHO classification classifies PSCCT as a morphological pattern of anaplastic thyroid carcinoma. It also lacks clearly defined therapy due to its rarity and has a poor prognosis with median survival being 9-12 months. We suggest that as fever, leukemoid reaction and hypercalcemia can be a paraneoplastic manifestation, one should think of PSCCT.
